# Parents’ Knowledge, Attitudes, and Practices Regarding Baby Walkers, Baby Car Seats, Oral Health, and Child Screen Time in Saudi Arabia: A Cross-Sectional Study

**DOI:** 10.7759/cureus.52464

**Published:** 2024-01-17

**Authors:** Rawan G Algethami, Shadi A Tamur, Rawan M Alsofyani, Hanan H Alfaifi, Faris M Qattan, Mazen S Alharthi, Maryam S Aljaid, Sultan A Almalki, Abdullah M Khayat, Ahmad S Alzahrani, Eman A Khalifa, Anawr M Shams

**Affiliations:** 1 Medicine and Surgery, College of Medicine, Taif University, Taif, SAU; 2 Pediatrics, College of Medicine, Taif University, Taif, SAU; 3 Medicine, College of Medicine, Taif University, Taif, SAU; 4 Microbiology and Parasitology, College of Medicine, Taif University, Taif, SAU; 5 Pharmacology, College of Medicine, Taif University, Taif, SAU

**Keywords:** knowledge, injury prevention, oral health, baby car seats, baby walkers, pediatric health

## Abstract

Background

Awareness of age-appropriate milestones and developmental stages is crucial for parents to identify any potential delays or concerns early on and seek appropriate interventions. This study aimed to assess the knowledge, attitudes, and practices of caregivers in Saudi Arabia regarding baby walkers, baby car seats, early dental visits, and screen time for young children.

Methods

A cross-sectional survey was conducted among parents in Saudi Arabia using a structured questionnaire. A convenience and snowball sampling method was employed to recruit participants from various regions of the country. The questionnaire aimed to assess parents’ knowledge regarding the recommended use of baby walkers and baby car seats, their awareness of the importance of early dental visits, and their understanding of appropriate screen time guidelines. Additionally, the survey explored parents’ practices toward these recommendations. Descriptive statistics were used to analyze the data, and associations between variables were examined using the chi-squared test.

Results

A total of 1318 participants were included. The analysis revealed that the majority of the participants (n=1066,81.3%) use a baby walker, while only (n=292,22.3%) consider that they should never be used. Overall, (n=388,29.6%) of the participants never used a car seat for their infants or children. In terms of early childhood dental visits, approximately (n=518,39.5%) of the participants reported actually taking their child to the dentist within the recommended timeframe. Regarding screen time for children, (n=148,11.3%) of the participants reported that their children spend >5 hours daily in front of the screen.

Conclusions

Raising parents’ awareness about recent childcare recommendations and safe practices is crucial for promoting optimal child development, preventing health problems, facilitating evidence-based decision-making, reducing risks, enhancing parental confidence and empowerment, and nurturing positive parent-child relationships.

## Introduction

Baby walkers are widely used devices designed to assist infants in their early attempts at independent mobility. They are made up of a seat suspended within a frame and equipped with wheels that enable babies to move around while seated [1,2]. The underlying concept is that the walker’s support improves the baby’s ability to move and develops their leg muscles, leading to earlier walking skills. Despite the fact that walkers may provide temporary entertainment and perceived benefits, such as accelerated walking skills, a growing body of scientific evidence has identified potential drawbacks and risks associated with their use, commonly, head and neck injuries from falling down the stairs, risk of burning, poisoning, and motor delay, all are considerable threats [3]. As a result, several countries have implemented regulations or even banned the sale and use of baby walkers [1,4]. Furthermore, healthcare professionals and child development experts consistently recommend alternative methods to support infants in their motor development. These methods include supervised floor play, tummy time, and age-appropriate toys that encourage crawling and independent exploration [5]. 

Baby car seats (BCSs) play a crucial role in safeguarding infants and young children while traveling in motor vehicles [6]. They are specifically engineered to provide optimal protection and support, thereby reducing the risk of severe injuries in the event of a car crash [7]. Scientific evidence consistently supports the notion that BCSs significantly enhance safety during road travel [8]. These seats are designed to minimize the forces exerted on an infant’s delicate body by distributing impact energy and reducing the risk of head and neck injuries. The inclusion of harness systems and side-impact protection features in modern car seats further contributes to their effectiveness in safeguarding young passengers [9].

Early childhood dental visits, starting as early as the eruption of the first tooth or by the age of one, provide an invaluable opportunity for parents and caregivers to establish a solid foundation of good oral health practices in children [10]. Therefore, implementing these early dental visits can significantly improve oral health outcomes in children [11]. Regular dental visits at an early age allow dental professionals to monitor and assess the child’s oral health status. They can also provide preventive measures such as fluoride treatments and dental sealants, which help protect against tooth decay. In addition, early detection of dental problems, such as cavities, malocclusions, or developmental abnormalities, enables prompt intervention and appropriate treatment, reducing the severity and potential complications of these conditions [12]. Parents can also gain knowledge about age-specific oral health concerns and receive guidance on appropriate dietary choices, oral hygiene techniques, and oral injury prevention. Scientific evidence consistently suggests that early dental visits are associated with better long-term oral health outcomes in children [11,13]. The combination of regular preventive dental care, early intervention, and parental education significantly reduces the risk of dental diseases, promotes proper dental development, and establishes a foundation for a lifetime of good oral health habits [14].

In today’s digital age, young children are frequently exposed to screens, including televisions, computers, smartphones, and tablets. However, excessive screen time during early childhood has been associated with potential negative effects on cognitive development [15]. A growing body of evidence suggests that excessive screen exposure may hinder language development, attention span, problem-solving skills, and executive functions [16,17]. Therefore, understanding the scientific research on screen time is crucial for parents, caregivers, and educators to make informed decisions. It is worth noting that reduced face-to-face interactions and limited exposure to nonverbal cues may have an impact on young children’s ability to develop and comprehend social skills, empathy, and emotional regulation [18]. Furthermore, excessive media exposure may increase the risk of developing socioemotional disorders such as aggression, anxiety, and decreased self-regulation [16].

Childcare is an essential aspect that plays a crucial role in providing a safe and nurturing environment for children to grow and develop. The quality of childcare services has a significant impact on children’s physical, emotional, and cognitive development. Health authorities have the main responsibility of promoting and ensuring children’s normal development by developing guidelines and recommendations to promote child health and safety [19]. Therefore, it is crucial to gain a comprehensive understanding of caregivers’ knowledge, awareness, attitudes, and behaviors regarding their children’s safety, oral health, and screen time habits. This research aimed to assess the knowledge, attitudes, and practices of caregivers in Saudi Arabia regarding baby walkers, BCSs, early dental visits, and screen time for young children. Accordingly, our research will provide valuable insights to identify potential gaps in knowledge and areas that require further education and intervention. The findings will help develop targeted educational programs, guidelines, and interventions to promote safe and healthy practices among caregivers in Saudi Arabia, ultimately enhancing the well-being and development of young children in these critical areas.

This article was previously posted to the medRxiv preprint server in September 2023.

## Materials and methods

A cross-sectional study was conducted to collect data on parents’ knowledge, attitudes, and practices regarding baby walkers, BCSs, early dental visits, and screen time for young children in Saudi Arabia. The study duration was 12 months. The data was collected using an online questionnaire. All normal, healthy Saudi parents or parents residing in Saudi Arabia of any age and gender with stable mental conditions and having children over the age of 13 were eligible to participate in this study. However, parents with preexisting mental conditions, parents with children below or equal to 13 years of age, and parents living outside Saudi Arabia were excluded from the study.

A mixed sampling technique, combining convenience and snowball sampling, was employed to select a representative sample of parents in Saudi Arabia. In the first stage, different regions were randomly selected. In the second stage, cities or towns within the selected regions were chosen. Five data collectors were assigned for data collection purposes, one for each region. Prior to the main data collection, a pilot study was conducted to ensure the questionnaire’s reliability and validity. The pilot study involved a small sample of parents or caregivers who were similar to the target population. Feedback from the pilot study participants was carefully considered to refine and improve the questionnaire. Three experienced senior faculty members from the institution, including a biostatistician, were involved in the questionnaire’s validation process.

The questionnaire, which is included in the Appendices section, was divided into sections to assess caregivers’ knowledge, attitudes, and practices regarding baby walkers, BCSs, early dental visits, and screen time. The first section of the questionnaire provided brief information about the study’s objectives, requirements, and potential benefits, followed by an informed consent statement. The online questionnaire was hosted on a secure online platform (Google Forms), allowing participants to complete it at their convenience.

Ethical considerations were thoroughly addressed throughout the study. Ethical approval was obtained from the Institutional Review Board (IRB) before commencing the research (the Scientific Research Ethics Committee at Taif University issued approval number 44-343). Informed consent was obtained from all participants prior to their participation in the online questionnaire. The confidentiality and privacy of participants’ information were strictly maintained, and any identifiable information was anonymized and securely stored.

Descriptive statistics were used to summarize the knowledge and practices of parents in Saudi Arabia regarding baby walkers, BCSs, oral health, and child screen time. Frequency distributions and percentages were computed to present the prevalence of specific knowledge levels, attitudes, and practices among the participants. Inferential statistics, such as chi-squared tests, were used to examine associations and relationships between variables, such as the relationship between parents’ knowledge about baby walkers and their sociodemographic characteristics. All statistical analyses were conducted using the Statistical Package for the Social Sciences software (SPSS), Version 25 (IBM Corp., Armonk, USA), with a p-value of less than 0.05 indicating statistical significance.

## Results

A total of 1384 invitations for participation were sent, of which 1318 participants gave consent and met the eligibility criteria for participation. The sociodemographic characteristics of the participants revealed that the highest percentage (n=300, 22.9%) were from the Makkah region, followed by the Eastern Province (n=211, 16.1%), Aseer (n=165, 12.6%), Al-Jouf (n=152, 11.6%), and Riyadh (n=151, 11.5%). In terms of age, 417 participants (31.8%) belonged to the age group of 36-45 years. The majority of the participants (n=911, 69.4%) were females. Furthermore, 982 participants (74.8%) had a university level of education, 1189 (90.6%) were married, and 402 (30.6%) had an income ranging from 6000 to 10000 Saudi Riyals (Table 1).

**Table 1:** Sociodemographic characteristics of the participants

		N	%
Region	Al-Baha	71	5.4
	Al-Jouf	152	11.6
	Al-Qassim	9	0.7
	Aseer	165	12.6
	Eastern Province	211	16.1
	Hail	8	0.6
	Jazan	103	7.9
	Makkah	300	22.9
	Medinah	91	6.9
	Northern borders	14	1.1
	Riyadh	151	11.5
	Tabuk	36	2.7
	Najran	1	0.1
Age (years)	<18	15	1.1
	18–25	213	16.2
	26–35	345	26.3
	36–45	417	31.8
	46–55	265	20.2
	>55	57	4.3
Gender	Female	911	69.4
	Male	401	30.6
Education	No primary education	10	0.8
	Primary	17	1.3
	Middle	44	3.4
	Secondary	259	19.7
	University	982	74.8
Marital status	Married	1189	90.6
	Divorced	75	5.7
	Widow	48	3.7
Monthly income (Saudi Riyals)	<5000	212	16.2
	6000–10000	402	30.6
	11000–15000	364	27.7
	>16000	334	25.5

The analysis revealed that 292 participants (22.3%) agreed that baby walkers should never be used, while the majority (n=1020, 77.7%) believed they should be used. The most common sources of this information were family and friends (n=362, 27.6%), followed by the Internet and medical personnel (n=220, 16.8%). Among the participants, (n=1066, 81.3%) reported using baby walkers, with the most common reason being to strengthen the baby’s leg muscles (n=583, 54.7%), followed by keeping the child busy and entertained (n=475, 44.6%), being able to do housework (n=324, 30.4%), and encouraging early walking (n=324, 30.4%). On the contrary, the most common reason for not using baby walkers was the belief that they are unnecessary (n=85, 34.6%), followed by complete dissatisfaction (n=77, 31.3%) and concerns regarding injuries (n=51, 20.7%) (Table 2).

**Table 2:** Knowledge and practices related to baby walkers

		N	%
Baby walkers should never be used	No	1020	77.7
	Yes	292	22.3
Source of information	Family and friends	362	27.6
	Internet	296	22.6
	Medical personnel (doctors, nurses, etc.)	220	16.8
	Social media	187	14.3
	Books	53	4.0
	Other	194	14.8
Using baby walkers	No	246	18.8
	Yes	1066	81.3
Reasons for using (n = 1066)	To make the child walk early	324	30.4
	To keep the child busy and entertained	475	44.6
	To strengthen the baby’s leg muscles	583	54.7
	To be able to do housework	324	30.4
	Other	64	6.0
Reasons for not using (n = 246)	Financial reasons	14	5.7
	Social/cultural reasons	20	8.1
	Delayed start of walking	37	15.0
	Complete dissatisfaction	77	31.3
	Cause harm to the male organs of their offspring	23	9.3
	Unnecessary	85	34.6
	Associated with injuries	51	20.7
	It was not suggested by the pediatrician	38	15.4

The findings on knowledge and practices related to BCSs are summarized in Table 3. A total of 812 participants (61.9%) agreed on the use of BCSs from birth to the age of 13 years. The primary sources of this information were the Internet (n=329, 25.1%), family and friends (n=245, 18.7%), and medical personnel (n=223, 17%). Among the participants, 924 (70.4%) reported using BCSs for their children. The most common reason for using these seats was to ensure child safety (n=865, 93.6%), followed by the desire to protect against irregularities (n=285, 30.8%), and adherence to traffic regulations (n=198, 21.4%). On the contrary, the most common reason for not using BCSs was the perception that they are not seen as important for babies (n=112, 28.9%), followed by child refusal (n=111, 28.6%).

**Table 3:** Knowledge and practices related to baby car seats

		N	%
Baby car seats should be used from birth to the age of 13 years	No	500	38.1
	Yes	812	61.9
Source of information	Family and friends	245	18.7
	Internet	329	25.1
	Medical personnel (doctors, nurses, etc.)	223	17.0
	Social media	221	16.8
	Books	51	3.9
	Other	243	18.5
Using baby car seats for your children	No	388	29.6
	Yes	924	70.4
Reasons for using baby car seats	Safety	865	93.6
	To protect against irregularities	285	30.8
	Due to traffic regulations	198	21.4
	Other	47	5.1
Reasons for not using baby car seats	Child’s fear of sitting in the seat	51	13.1
	I do not see the importance of that	112	28.9
	Child refusal	111	28.6
	The child is very small	56	14.4
	Others	131	33.8

The findings on knowledge and practices related to early childhood dental visits are summarized in Table 4. It was found that approximately (n=796, 60.7%) of the participants agreed on the necessity of a dental visit within six months of the first tooth eruption or when the child reaches one year old. The primary sources of this information were medical personnel (n=387, 29.5%), followed by the Internet (n=243, 18.5%), and family and friends (n=241,18.4%). Surprisingly, only (n=518, 39.5%) of the participants reported actually taking their child to the dentist within the recommended timeframe. Conversely, the most common reason for not taking the child to the dentist early was the belief that it was unnecessary (n=442, 55.7%), followed by a lack of complete conviction (n=153, 19.3%). Regarding the frequency of dental visits for children, it was found that approximately (n=766, 58.4%) of the participants only sought dental care when there were symptoms, while (n=256, 19.5%) visited every six months and (n=211, 16.1%) visited annually.

**Table 4:** Knowledge and practices related to early childhood dental visits

		N	%
A dental visit is necessary within six months of the first tooth eruption or when the child reaches one year old	No	516	39.3
	Yes	796	60.7
Source of information	Family and friends	241	18.4
	Internet	243	18.5
	Medical personnel (doctors, nurses, etc.)	387	29.5
	Social media	134	10.2
	Books	41	3.1
	Other	266	20.3
Taking the child to the dentist within six months of the first tooth eruption or when the child reaches one year old	No	794	60.5
	Yes	518	39.5
Reasons for not taking the child to the dentist (n = 794)	Financial reasons	75	9.4
	Social/cultural reasons	35	4.4
	Lack of complete conviction	153	19.3
	Unnecessary	442	55.7
	Other	195	24.6
The frequency of dental visits for children	Once every 6 months	256	19.5
	Once a year	211	16.1
	Only when there are symptoms (pain, abscess, etc.)	766	58.4
	Never	79	6.0

The findings on knowledge and practices related to screen time for children are summarized in Table 5. It was found that the majority of the participants (n=1097, 83.6%) agreed on the correct recommendation for screen time for children. The primary sources of this information were the Internet (n=322, 24.5%), followed by medical personnel (n=304, 23.2%) and social media (n=255, 19.4%). Approximately (n=853, 65%) of the participants agreed to apply the recommended screen time for their children. In terms of the average screen time for children, (n=846, 64.5%) of the participants reported that their children spent 2-3 h per day on screens, while (n=318, 24.2%) reported 4-5 h per day, and (n=148, 11.3%)reported more than 5 h per day. Regarding the person with whom children spent most of their time, it was found that parents accounted for the majority (n=1087, 82.9%), followed by grandfathers (n=121, 9.2%), and babysitters or housemaids (n=82, 6.3%). Notably, children who spent most of their time with babysitters or housemaids had a significantly higher screen time of more than 5 h per day than those who spent most of their time with parents. However, children who spent most of their time with parents had significantly higher screen time of 2-3 h than others (p < 0.001).

**Table 5:** Knowledge and practices related to television and smart device screens for children

		N	%
Children are not allowed to watch television and smart device screens from birth until 18 months of age. From 18 months to 24 months, a limited screen time of no more than 1 h per day with a parent or caregiver is allowed. In the preschool stage, screen time should not exceed 1 h per day with a parent or caregiver. From 5 to 18 years of age, the screen time should not exceed 2 h per day.	No	215	16.4
	Yes	1097	83.6
Source of information	Family and friends	164	12.5
	Medical personnel	304	23.2
	Internet	322	24.5
	Social media	255	19.4
	Books	64	4.9
	Other	203	15.5
Applying the screen time recommendation to children	No	459	35.0
	Yes	853	65.0
Reasons for not following the screen time recommendation (n = 459)	Financial reasons	9	2.0
	Social/cultural reasons	109	23.7
	Complete dissatisfaction	98	21.4
	Unnecessary	82	17.9
	Others	215	46.8
The number of hours your child spends watching screens	2–3 h a day	846	64.5
	4–5 h a day	318	24.2
	More than 5 h a day	148	11.3
Person with whom children spend most of their time	Parents	1,087	82.9
	Grandfathers or relatives	121	9.2
	Babysitters or housemaids	82	6.3
	In the nursery	22	1.7

The relationship between knowledge related to baby walkers, BCSs, dental visits, and screen time, and sociodemographic characteristics is summarized in Table 6. It was found that participants with or without primary education had significantly higher correct responses to recommendations for baby walker use than others (p = 0.001). Additionally, participants who were married or divorced showed a comparatively positive response to recommendations for BCS use (p = 0.019). However, no statistically significant relationship was observed between responses to recommendations for early childhood dental visits and any sociodemographic characteristics (p > 0.05). Meanwhile, it was observed that female participants gave significantly more correct responses to recommendations for screen time than males (p = 0.008).

**Table 6:** Knowledge related to baby walkers, baby car seats, dental visit, and screen time and their relationship with sociodemographic characteristics

			Baby walkers			Baby car seats			Dental visit within six months of the appearance of the first tooth			Screen time		
			Correct	Wrong	P value	Correct	Wrong	P value	Correct	Wrong	P value	Correct	Wrong	P value
Age	<18	N	4	11	0.985	9	6	0.084	10	5	0.055	9	6	0.109
		%	26.7	73.3		60.0	40.0		66.7	33.3		60.0	40.0	
	18-25	N	45	168		119	94		144	69		181	32	
		%	21.1	78.9		55.9	44.1		67.6	32.4		85.0	15.0	
	26-35	N	79	266		223	122		193	152		295	50	
		%	22.9	77.1		64.6	35.4		55.9	44.1		85.5	14.5	
	36-45	N	93	324		275	142		249	168		341	76	
		%	22.3	77.7		65.9	34.1		59.7	40.3		81.8	18.2	
	46-55	N	57	208		154	111		170	95		225	40	
		%	21.5	78.5		58.1	41.9		64.2	35.8		84.9	15.1	
	>55	N	14	43		32	25		30	27		46	11	
		%	24.6	75.4		56.1	43.9		52.6	47.4		80.7	19.3	
Gender	Female	N	206	705	0.640	561	350	0.728	555	356	0.779	778	133	0.008
		%	22.6	77.4		61.6	38.4		60.9	39.1		85.4	14.6	
	Male	N	86	315		251	150		241	160		319	82	
		%	21.4	78.6		62.6	37.4		60.1	39.9		79.6	20.4	
Educational level	No primary education	N	7	3	0.001	8	2	0.802	9	1	0.316	8	2	0.976
		%	70.0	30.0		80.0	20.0		90.0	10.0		80.0	20.0	
	Primary	N	7	10		11	6		9	8		15	2	
		%	41.2	58.8		64.7	35.3		52.9	47.1		88.2	11.8	
	Middle	N	11	33		27	17		24	20		36	8	
		%	25.0	75.0		61.4	38.6		54.5	45.5		81.8	18.2	
	Secondary	N	59	200		157	102		157	102		217	42	
		%	22.8	77.2		60.6	39.4		60.6	39.4		83.8	16.2	
	University	N	208	774		609	373		597	385		821	161	
		%	21.2	78.8		62.0	38.0		60.8	39.2		83.6	16.4	
Marital Status	Married	N	259	930	0.408	740	449	0.019	712	477	0.135	998	191	0.119
		%	21.8	78.2		62.2	37.8		59.9	40.1		83.9	16.1	
	Divorced	N	21	54		51	24		49	26		64	11	
		%	28.0	72.0		68.0	32.0		65.3	34.7		85.3	14.7	
	Widow	N	12	36		21	27		35	13		35	13	
		%	25.0	75.0		43.8	56.3		72.9	27.1		72.9	27.1	
Family monthly income	<5000	N	48	164	0.071	128	84	0.782	130	82	0.575	173	39	0.617
		%	22.6	77.4		60.4	39.6		61.3	38.7		81.6	18.4	
	6,000-10,000	N	102	300		257	145		238	164		342	60	
		%	25.4	74.6		63.9	36.1		59.2	40.8		85.1	14.9	
	11,000-15,000	N	84	280		222	142		231	133		307	57	
		%	23.1	76.9		61.0	39.0		63.5	36.5		84.3	15.7	
	>16,000	N	58	276		205	129		197	137		275	59	
		%	17.4	82.6		61.4	38.6		59.0	41.0		82.3	17.7	

## Discussion

The findings of this study revealed that less than one-fourth of the participants agreed that baby walkers should never be used. There is insufficient evidence to support the notion that baby walkers are beneficial for a child’s development [1,20]. They do not contribute to the development of motor skills or promote early walking in newborns. Babies are unable to practice important motor skills like pulling up, creeping, and crawling while using a walker [21]. However, approximately 81.3% of the participants reported using walkers for their children, with the most common reason being to strengthen the baby’s leg muscles. A previous study conducted in Saudi Arabia reported a similar prevalence of baby walker use, with the most common reason being to make the child walk earlier [22]. 

In a study conducted in the United States by Smith et al. on baby walker-related injuries, the authors found that 59% of parents were aware of the potential hazards posed by baby walkers before any injury occurred to their child. Despite having this understanding, 45% of families chose to keep the walker after the injury, and 32% used it once more, either for the child who was injured or for a different child [23].

Over time, false beliefs among parents that baby walkers promote early walking and are typically safe have contributed to common misconceptions among baby walker users. Through measuring parents’ knowledge levels, we discovered these two misconceptions. The vast majority of respondents believed these misconceptions to be true. Furthermore, baby walker users demonstrated lower levels of awareness than nonusers. A previous study reported similar findings [24]. However, existing literature disproves all these misconceptions [25]. Our research findings support the hypothesis that a lack of parental education and experience with baby walkers contributes to higher usage.

Child passengers are significantly safer when car seats are used properly. In our study, the majority of the parents (70.4%) reported using baby seats in the car. Another study conducted in Unaizah City reported that 55.1% of parents used BCSs. The main reasons for not using BCSs in our study were that they were perceived as unimportant for the baby, followed by child refusal.

Other studies have identified several barriers. A study conducted in China found that the lack of awareness and laws requiring the use of child safety seats were perceived as barriers to their use [26]. Although we did not find an association in our study between the parent’s education level and the use of BCSs, a study from Slovenia reported that mothers with lower education were more likely to not use BCSs during short trips [27].

It is common for children to resist using BCSs and rely on adults to properly restrain them in the car. It is crucial for adults to know how to properly restrain children who may not cooperate with using BCSs [28]. Children are particularly vulnerable to injury in traffic accidents due to their physical characteristics and cognitive development. The risk of injury is significantly higher if they are not properly restrained in the car. BCS has been proven effective in reducing fatalities and serious injuries caused by car accidents for a long time [29]. Age- and height-appropriate BCSs and seat belts are recommended by both the World Health Organization and guidelines in industrialized countries [28]. Counseling parents in primary healthcare centers about the importance of using BCSs with their children, along with education about passenger safety in schools, has been shown to increase compliance in the short term.

Early detection of dental diseases is crucial, as it leads to better cooperation from children and reduces costs for parents by shortening treatment times and minimizing missed workdays. Preschoolers heavily rely on their parents to provide for their oral healthcare needs, highlighting the importance of starting to protect their oral health at an early age. The findings of our study revealed that 60.7% of parents agreed on the necessity of early childhood dental visits within six months of the first tooth eruption or when the child reaches one year old. However, only 39.5% reported actually taking their children for such visits. The most common reason for not taking the child to a dental visit early was the belief that it was unnecessary. 

In a study conducted by Trinh et al. in Australia, a significant proportion of caregivers lacked awareness of the significance of early dental appointments for their offspring. The authors reported that only 22.4% of parents knew that the initial dental checkup should be conducted at 1 year of age or earlier [30]. Common reasons cited included lack of time, inadequate knowledge of oral hygiene practices, job stress, and the growing number of nuclear families and working parents [31]. Caregivers with inaccurate perceptions about the importance of primary teeth are less likely to seek preventive dental visits for their children [32]. It is worth noting that positive oral health knowledge and attitudes may not always translate into positive behaviors [33,34]. Therefore, it is essential for doctors to train and motivate parents to take their children to the dentist at an early age and guide them to perform oral hygiene practices properly and effectively.

Children’s daily lives are increasingly dominated by screens due to their accessibility, engaging nature, and frequent use [35]. While there may be some positive effects of screen time for kids, research has linked long-term exposure to negative developmental health outcomes, such as obesity, behavioral problems, emotional regulation issues [8,9], speech delays, and reduced executive functioning [36-38]. The findings of this study revealed that the majority of the parents (83.6%) agreed with the screen time recommendations for children. However, only 65% reported adhering to these recommendations for their own children. 

The authors of a study conducted in the United States found that 62.2% of mothers exhibited awareness of screen time recommendations put forth by the American Academy of Pediatrics. However, only 46.1% demonstrated the ability to accurately recall and cite these recommendations [39]. Furthermore, children who did not spend most of their time with their parents had significantly higher screen time, as reported by their parents. Notably, young children under the age of five benefit most from face-to-face, in-depth interactions with adults. When given the choice, children prefer engaging in interactive activities such as conversation, play, or reading rather than staring at a screen [40]. Considering the pervasive use of electronic devices, it becomes necessary to focus not only on the total amount of screen time but also on the type of screen-based activities in which children engage. Further extensive multilevel longitudinal research is needed to explore the various aspects of screen time. Clinicians and other service providers should pay special attention to children who exhibit compulsive demands for screen time, particularly those using smartphones. It is important to consider the influence of a parent’s own social media use and behaviors alongside those of their child [41]. In a study conducted in Brazil by Goncalves et al., a significant correlation was identified between the screen time of parents and that of their children, underscoring the significance of parental role modeling [42]. Future studies should incorporate observational measurements of parent-child interactions to confirm these results.

While our study benefits from a large sample size, some limitations should be acknowledged before generalizing the findings. First, the reliance on self-report measures may introduce potential biases. Parents’ over-reporting responses may have been influenced by social desirability bias, leading to an over-reporting of positive practices or an underreporting of negative practices. This limitation could have an impact on the validity and reliability of the collected data. Second, the study was cross-sectional in design, capturing data at a specific time point. This design limitation restricted our ability to establish causal relationships between parents’ knowledge and practices and their children’s outcomes. Longitudinal studies would provide more robust evidence of the impact of these factors over time. Third, our study focused solely on parents’ knowledge and practices, neglecting other influential factors. These unaccounted variables could have confounded the results and limited the comprehensive understanding of the topic.

## Conclusions

The findings of this study indicate that while most parents were aware of recommendations regarding baby walkers, BCSs, early dental visits, and screen time for young children, their actual practice did not align with these recommendations. It is crucial to raise parents’ awareness of recent childcare recommendations and safe practices in order to promote child development and prevent health problems. When parents are aware of the current childcare recommendations, they can provide the necessary support and guidance for their child’s development. Furthermore, acquiring knowledge about safe practices can significantly contribute to the prevention of health problems in children. Parents who are well-informed about proper nutrition, hygiene, immunization, and safety measures can create a healthy environment that minimizes the risk of accidents, injuries, and the spread of infections or diseases.

## Appendices

Questionnaire 
 

Part A

1.     Are you willing to participate in this research?

A.    Yes

B.    No

2.     Do you have children under the age of 13?

A.    Yes

B.    No

3.     Are you a Saudi citizen residing in Saudi Arabia?

A.    Yes

B.    No

4.     Please specify the region of residence if you are Saudi citizen residing in Saudi Arabia.

A.    Riyadh

B.    Makkah Al-Mukarramah

C.     Al-Madinah Al-Munawwarah

D.    Al-Qassim

E.     Eastern Province

F.     Aseer

G.    Tabuk

H.    Hail

I.      Northern Borders

J.      Al-Baha

K.    Jazan

L.     Najran

M.   Al-Jawf

5.     What is your age?

A.    Under 18 years old

B.    18-25 years old

C.     26-35 years old

D.    36-45 years old

E.     46-55 years old

F.     Over 55 years old

6.     Gender:

A.    Male

B.    Female

7.     Educational level:

1.     Illiterate

2.     Primary

3.     Intermediate

4.     Secondary

5.     University

8.     Marital status:

A.    Married

B.    Divorced

C.     Widowed

9.     Monthly family income (in Saudi Riyals):

A.    Less than 5,000 

B.    6,000-10,000

C.     11,000-15,000

D.    More than 16,000

Part B

1.     Baby walkers should never be used?

A.    Strongly agree

B.    Agree

C.     Neutral

D.    Disagree

E.     Strongly disagree.

2.     Please specify the source of your information:

A.    Internet

B.    Social media

C.     Medical staff (doctors, nurses, etc.)

D.    Family and friends

E.     Books

F.     Other: ___________

3.     Do you use baby walkers for your children?

A.    Yes

B.    No

4.     If you answered ‘Yes;, please specify the reason for its use:

A.    To be able to do housework

B.    To make the baby walk earlier

C.     To keep the baby occupied and entertained To make the baby’s legs stronger

D.    Other reasons

5.     If you answered no, please specify the reason for your lack of compliance:

A.    Financial reasons

B.    Social / cultural reasons

C.     Lack of complete conviction

D.    Delay beginning to walk

E.     Give harm to their sons’ genital organs 

F.     Unnecessary

G.    Associated with injuries

H.    Paediatrician did not suggest

Part C

1.     Car seats should be used for children from birth up to 12  years old?

A.    Strongly agree

B.    Agree

C.     Neutral

D.    Disagree

E.     Strongly disagree.

2.     Please specify the source of your information:

A.    Internet

B.    Social media

C.     Medical staff (doctors, nurses, etc.)

D.    Family and friends

E.     Books

F.     Other:

3.     Do you use special car seats for children?

A.    Yes

B.    No

4.     If you answered ‘Yes;, please specify the reason for its use:

A.    Security 

B.    To protect from irregularities

C.     Due to motor traffic regulations

D.    Other

5.     If you answered NO, please specify the reason for your lack of compliance:

A.    Fear of the child sitting in the seat              

B.    I do not see it is important               

C.     The child refused                  

D.    The child is too young                      

E.     Others

Part D

1.     A dentist visit is necessary within six months of the appearance of the first tooth or by the first birthday?

A.    Strongly agree

B.    Agree

C.     Neutral

D.    Disagree

E.     Strongly disagree.

2.     Please specify the source of your information:

A.    Internet

B.    Social media

C.     Medical staff (doctors, nurses, etc.)

D.    Family and friends

E.     Books

F.     Other:

3.     Did you take your child to a dentist within six months of the appearance of the first tooth or by his/her first birthday?

A.    Yes

B.    No

4.     If you answered No what was the reason?

A.    Financial reasons

B.    Social / cultural reasons

C.     Lack of complete conviction

D.    Unnecessary

E.     Other

5.     How often do you take your child to the dentist?

A.    Once in 6 months

B.    Once a year

C.     Only when there are symptoms (pain, abscess, etc.)

D.    Never

Part E

1.     In preschool Children, Age 5 to 18 years old, “Screen time” it should not exceed two hours per day.

A.    Strongly agree

B.    Agree

C.     Neutral

D.    Disagree

E.     Strongly disagree.

2.     Please specify the source of your information:

A.    Internet

B.    Social media

C.     Medical staff (doctors, nurses, etc.)

D.    Family and friends

E.     Books

F.     Other:

3.     Do you apply this to your children?

A.    Yes

B.    No

4.     If you answered NO what is the reason?

A.    Financial reasons

B.    Social / cultural reasons

C.     Lack of complete conviction

D.    Unnecessary

E.     Other

5.     Please specify the number of hours your child spends watching screens:

A.    Two to three hours per day

B.    Four to five hours per day

C.     More than five hours per day

6.     Who does your child spend most of their time with?

A.    Parents

B.    Grandparent or relative

C.    Nanny or domestic worker

D.    In daycare
